# Johann Gregor Mendel: the victory of statistics over human imagination

**DOI:** 10.1038/s41431-023-01303-1

**Published:** 2023-02-09

**Authors:** Martina Raudenska, Tomas Vicar, Jaromir Gumulec, Michal Masarik

**Affiliations:** 1 Department of Physiology, Faculty of Medicine, Masaryk University/Kamenice 5, CZ-625 00 Brno, Czech Republic; 2 Department of Pathological Physiology, Faculty of Medicine, Masaryk University/Kamenice 5, CZ-625 00 Brno, Czech Republic; 3 Department of Biomedical Engineering, Faculty of Electrical Engineering and Communication, Brno University of Technology, Technicka 3058/10, Brno, Czech Republic; 4 BIOCEV, First Faculty of Medicine, Charles University, Prumyslova 595, CZ-252 50 Vestec, Czech Republic

**Keywords:** Behavioural genetics, Genetics research

## Abstract

In 2022, we celebrated 200 years since the birth of Johann Gregor Mendel. Although his contributions to science went unrecognized during his lifetime, Mendel not only described the principles of monogenic inheritance but also pioneered the modern way of doing science based on precise experimental data acquisition and evaluation. Novel statistical and algorithmic approaches are now at the center of scientific work, showing that work that is considered marginal in one era can become a mainstream research approach in the next era. The onset of data-driven science caused a shift from hypothesis-testing to hypothesis-generating approaches in science. Mendel is remembered here as a promoter of this approach, and the benefits of big data and statistical approaches are discussed.

## Johann Gregor Mendel and the birth of biological statistics

Mendel was born on 20 July 1822 in Hynčice (Heinzendorf), Moravia, which was then a province of the Austro-Hungarian Empire (now part of the Czech Republic). He was baptized Johann and took the name Gregor in 1843 when he entered the Augustinian Monastery of St. Thomas at Brno. The role of a monk extended far beyond the care of men’s souls at that time. Monasteries were centers of deep interest in science, culture, and trade. After his ordination, Mendel devoted himself to pastoral duties but found himself totally unsuitable for the job because of his shyness. Then, he started to teach in a secondary school in Znojmo (Znaim). However, he failed to gain his teacher’s certificate and was sent to the University of Vienna for further education. In Vienna, he studied several subjects, including physics under Professor Christian Doppler (the discoverer of the famous Doppler effect), chemistry, paleontology, and plant physiology with Franz Unger, who introduced him to the most recent scientific ideas, such as the newly developed cell theory [1]. During his studies, Mendel became an excellent experimenter and gained familiarity with the doctrine of discrete units (atoms and molecules) as the essence of physical and chemical processes. Mendel returned to Brno in 1853, and he continued in the position of the secondary school teacher despite lacking full qualifications. In 1856, he again failed the exam to become a qualified teacher. Abbot Cyril Napp rescued Mendel’s career by authorizing a program of experimental hybridization at the St. Thomas monastery.

At that time, growers and breeders were puzzled that they could not fully elucidate the rules of heredity. For generations, they had produced detailed records of features such as the characteristics of cattle, the color of flowers, or the number of kernels in an ear of maize, but the hereditary process still seemed to be random. Sometimes, characteristic features were missing for several generations and then reappeared, and sometimes they disappeared completely. To get to the bottom of these issues, Mendel decided that it was necessary to first isolate clearly defined phenotypic traits. He did not try to track all phenotypic traits at once but chose a few clearly defined features (characteristics such as plant height, flower color, or seed shape). Although he began his research using mice, he later switched to plants because his bishop (Anton Ernst Schaffgotsch) found studying animal sexuality offensive and inappropriate for the status of a monk. Hence, Mendel decided to use garden peas as his primary experimental model. Luckily, garden peas turned out to be a practical and suitable model, as they took up little space, were cheap, and produced offspring quickly. Between 1854 and 1856, Mendel cultivated and tested thousands of pea plants because he assumed that the more independent measurements he made, the more likely it would be that he could rule out random phenomena. His exhaustive study included tests of 34 varieties of garden peas for trait consistency over several generations. He eventually developed 22 varieties of pea plants with consistent characteristics. Mendel also repeated his experiment to verify his results [2]. Based on his famous experiments, he proposed that discrete units referred to as factors determine the appearance of a trait and that for each physical trait, each factor has two contributing forms. He analyzed the transmission of studied traits by using statistical models of probability. Thus, Mendel began to apply statistics in biological research, which was a pioneering approach. It must also be emphasized that Mendel discovered his laws without any hypotheses about the mechanism of heredity.

The fact that he did not try to discover the universal evolutionary laws and mechanisms of heredity was probably responsible for the failure of his work in the scientific world. Perhaps “Mendel’s way of thinking was more like a farmer’s than a biologist’s” [3]. Mendel was obviously considered an outsider by the scientific community. Reputable plant physiologist Carl Wilhelm von Nägeli, with whom Mendel had a long correspondence (from 1866 to 1873), was competent enough to understand the significance of Mendel’s work but did not give it much attention [1]. Moreover, he thought that Mendel’s conclusions were wrong. He obviously distrusted amateur scientists as he added the derogatory note “only empirical…. cannot be rationally proven” to his first letter to Mendel, as if experimentally discovered laws were somehow inferior to those based on rational thinking and imagination [4].

## Big data, why imagination is not enough

Nägeli’s position has not aged well. Today’s science is based on carefully validated experimental data, and a hypothesis is sometimes not put forth before obtaining valid experimental data; instead, a large amount of data can first be collected, and a hypothesis can be developed based on those data. Currently, it is completely impossible for scientists to review large datasets and evaluate their data themselves, as important patterns in these data are mainly unrecognizable by the human brain. The shift toward data-driven science caused a subsequent shift from hypothesis-testing to hypothesis-generating scientific approaches. The speed of data generation is increasing. The completion of the Human Genome Project took approximately 13 years and cost more than £2 billion. Today, next-generation sequencing (NGS) can produce a whole genome in 24 hours for a thousand pounds [4]. Rapid NGS allowed genome-wide association studies to be performed, in which thousands of genomes from people with or without a given disease can be considered. Consequently, an algorithm is needed to compare genomes, identify differences, and then determine which genes are linked to the disease without having to consider either candidate genes or genes in general, as changes in noncoding DNA may also be involved in a disease. While tracing the relationship between genotype and phenotype is relatively straightforward in the case of monogenic diseases, it is a pressing problem in the case of multifactorial diseases. Importantly, socially significant diseases such as cardiovascular diseases, diabetes mellitus, or oncologic diseases are almost all complex and are associated with tens or sometimes hundreds of genes or sections of noncoding DNA in combination with environmental and lifestyle factors, such as exercise, diet, or pollutant exposure.

One of the most difficult cases in tracing the link between genotype and phenotype is cancer. Cancer cells are evolving systems and can change their genetic background under selection pressure. Accordingly, finding the connection between such complex genetic background and the emergence of the cancer phenotype may be difficult and almost impossible without using computational methods. Intensive efforts to identify cancer-contributing factors lead to the generation of big data. Novel algorithmic methods such as artificial intelligence (AI) and machine learning (ML), which can be used to identify patterns and trends in the data that may not be intuitively evident, were introduced to enable scientists to process big data generated under international efforts such as the Pan-Cancer Analysis of Whole Genomes (PCAWG) and the International Cancer Genome Consortium (ICGC) projects. The ICGC project involves international large-scale cancer genome studies addressing 50 different clinically important cancer types and/or subtypes. More than 25,000 cancer patients were systematically studied at the genomic, epigenomic, and transcriptomic levels [5]. A major discovery arising from these big data was the confirmation of the significant complexity of cancer genomes. Despite sharing the same type of malign condition, each patient exhibits a unique set of mutations, single nucleotide polymorphisms (SNPs), or chromosomal changes. Such diversity in the genetic landscape can explain differences in treatment resistance and patient outcomes. Furthermore, the NGS of tumors has disproved the idea that tumors are a mass of genetically identical, cloned cells. On the contrary, one tumor can contain dozens of different cell types, each with different combinations of genetic changes conferring vulnerability to different drugs or treatment regimens [6]. On the other hand, NGS has also revealed some unexpected similarities between cancers. There can be tumors at different anatomical sites that share more in common with each other than with tumors at the same anatomical site. Consequently, some therapeutic drugs can be effective against histologically different cancers. For example, pembrolizumab (humanized monoclonal antibody that blocks the interaction between Programmed death-1 (PD-1) and its ligands) may be beneficial to any cancer patient harboring mismatch repair deficiency [7]. Despite the complexity of cancer, deriving meaningful clinical predictors from genomic or proteomic data can be achieved but requires sustainable updating of databases and comprehensive clinical characterization of tens of thousands of patients [8]. As these sample sizes are too large for any single agency or institution to collect, extensive international collaboration and data sharing is needed. It is also necessary to use supercomputing to tailor personalized treatments based on big data on the genome, proteome (sets of proteins that are expressed by cells or tissues at a certain time and physiological state), or interactome (whole set of molecular interactions in a particular cell) and the etiology of the disease because the scale of the data and the elucidation of possible connections will far exceed human imagination.

## (Do not) be afraid of black boxes

As Mendel had no idea of the existence of chromosomes or genes, he focused on the input and output data represented by the plant phenotypes. The mechanism of heredity was a black box to him. However, he correctly concluded that a specific unit factor exists for each trait (now known as an allele) and that each diploid individual has two of these unit factors, one inherited from each parent.

The development of diseases also represents a black box between the genotype and the resulting pathological phenotype. We are not yet able to understand all mechanisms involved in the production of phenotypes from genotypes, but we can make some sensible predictions based on the available data. Moreover, a good prediction model does not need to provide an understanding of the exact molecular mechanisms involved in developing specific phenotypes or disorders. Deep learning (DL), a subdomain of machine learning, significantly increases the capabilities of the relevant prediction models. The prediction model only provides output information based on the available input data. However, when there is a large amount of input data (such as a combination of genomic, proteomic, metabolomic, or patient clinical data, and theoretically many others), simple models may fail; preferably, deep learning-based methods can be used [9]. This approach has been successfully applied in various genomics applications, such as the prediction of responses to cancer therapy [10] or the inference of gene relationships from single-cell expression data [11]. In these applications, DL provided superior performance over previous methods, and we can expect increasing significance of DL as its performance increases with increasing amounts of training data [9]. Nevertheless, the flip side of well-classified neural networks is the nonintuitive interpretation of their architecture, a problem that is again referred to as a black box. This lack of straightforward interpretability in clinical applications is perceived as a disadvantage of DL. Understanding network decision making can convince a physician that a diagnosis is legitimate [12]. Multiple approaches have been adopted to make deep learning networks explainable. For instance, a “reverse-engineered” DL network was shown to aid in the identification of aggressive melanoma cancers based on imaging data: by creating in silico images of cells that had never been observed experimentally, the morphological features of aggressive melanoma cells were identified [13]. Similar approaches may also enable DL expansion into other fields of diagnosis.

## Big data and statistical approaches

The statistical approaches that were first implemented in Mendel’s study really paid off, as they have practical relevance for medicine and biological sciences. Bioinformatics has been one of the key scientific disciplines in the fight against COVID-19. These approaches allowed the successful inference of the genomic architecture of the SARS-CoV-2 virus, and in silico studies involving NGS, genome-wide association studies, and computer-aided drug design have been effectively applied in the fight against COVID-19 [14]. One of the best examples of the achievements of bioinformatics is found in the realm of HIV treatment. HIV is characterized by rapid mutation, which allows it to evade antiretroviral drugs. The standard process for predicting the level of resistance relies on known mutations conferring resistance to various antiretroviral therapies. However, ML methods can now be used to address this problem [15]. The design of new medicinal products is another benefit of algorithmic approaches. ML methods have been successfully introduced for the prediction of drug-target interactions, which was made possible by the large amounts of drug and target data available in existing databases [16]. The field of cancer genomics has perhaps seen the most exciting developments, mainly in relation to leukemia. Inclusive, multistage statistical models can accurately predict the likelihood of remission, relapse, and mortality in patients with leukemia. The comparison of long-term survival probabilities under different treatments can provide therapeutic decision support (available online) [8]. It is also possible to discriminate age-related clonal hematopoiesis from stages preceding acute myeloid leukemia many years before malignant transformation [17]. The application of bioinformatic computational approaches together with the whole-genome sequencing analysis of 2,658 cancers allowed the reconstruction of the life history and evolution of mutational processes and driver mutation sequences in 38 types of cancer [18].

The study of human cells as a system requires a near-complete list of all cellular components (genes, methylation, noncoding RNA, proteins, transcripts, organelles, etc.) and comprehensive maps (interactomes) of how these factors interact with each other to mediate cellular functions. An integrated strategy that could revolutionize genetic research lies in combining interactome big data with ML, which allows the mining of information hidden in data to identify the genetic models or networks that control various phenotypic traits [19]. In multi-omic settings (different proteomic, genomic data or knowledge about interactome are combined during this type of analysis), the power of DL has been demonstrated in many areas, including miRNA-encoding sequences identification and miRNA target gene prediction by DeepMirGene (miRNA precursor prediction algorithm) and DeepTarget (end-to-end machine learning framework for miRNA target prediction) [20, 21], the inference of target gene expression from the expression of landmark genes by D-GEX [22], and gene expression prediction based on histone modifications by DeepChrome [23]. Furthermore, DeepVariant can call genetic variation in aligned NGS read data [24], and the DeepFIGV model has successfully predicted locus-specific signals for alternative alleles from four epigenetic assays using only DNA sequences as the input [25]. In work closer to clinical applications, we see the predictive potential for survival analyses, as shown by DeepSurv, DeepHIT, or DeepOmix networks [26–28]. With the last tool, it is possible to extract relationships between survival and multi-omics data. These applications are supervised networks, and most of them use unified data; however, in most of these applications, DL significantly outperforms other methods.

## Deep learning limitations and future perspectives

Given the boom in biotechnological innovations that will lead to new high-throughput measurements and accelerate the generation of big data at the cellular and molecular levels (currently, the amounts of these data are increasing exponentially and are already too large and complex for visual investigation [29]), it can be expected that the importance of ML and DL to basic and applied life sciences will increase considerably in the future [30].

**Fig. 1:**
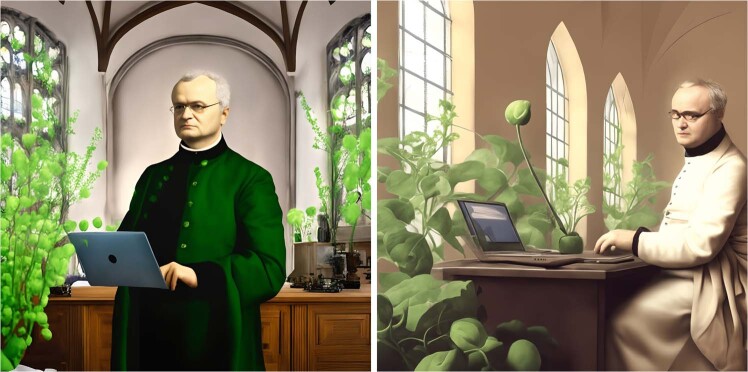
Demonstration of the possibilities of data processing using deep learning. Artificially generated images using keywords: „8k photograph of Johann Gregor Mendel in his gothic monastery laboratory with pea plants, working with deep learning on his computer“. Generated with Stable Diffusion 2.1.

Nevertheless, we need to be aware of the limitations of these methods. Their greatest success lies in their prediction accuracy; however, we are often more interested in discovering biological mechanisms than in black box prediction accuracy [31]. ML uses handcrafted features for prediction, while DL is typically applied to raw data, and feature extraction is performed by the model, which further decreases interpretability. If these tools have been available to Mendel, he would not have chosen the seven pea plant characteristics that he studied (height, pod shape, seed shape, pea color, and others) manually but would have relied on feature extraction via DL networks (for illustration see Fig. 1; this image was artificially generated by using a deep learning text-to-image model). For example, a computational approach would have helped Mendel to select features that are encoded in separate chromosomes and therefore always segregate independently. The discovery of crossing over and resulting recombination occurred approximately 100 years after Mendel’s experiments. However, Mendel was lucky; he chose characteristics encoded by genes that were located either far from each other on the same chromosome or on different chromosomes, which enabled the postulation of Mendel’s law of segregation.

ML (and especially DL) models are very successful in supervised tasks based on large amounts of labeled training data with a clearly defined input and output of the model; however, omics data include heterogeneous features of various types (numerical and categorical features and signals or images), and labels (e.g., clinical outcomes) include large amounts of randomness and noise, which makes the application of DL problematic [32, 33]. Nevertheless, this approach can still handle multimodal data, albeit at the cost of lower interpretability [30, 34]. Another great advantage of DL methods is end-to-end learning: DL does not require a feature extraction preprocessing step, which is time consuming and error prone because of the large variety of multi-omic data sources [30]. Even though the most successful applications of DL in multi-omics are supervised, generative models, such as generative adversarial networks (GANs), the use of two neural networks, comparing them against each other, has shown the potential of these models in various fields [31, 34].

The greatest successes of DL have been achieved in text and image processing, where the largest amounts of data are available. With a sufficient amount of data and model size, DL models can be developed from single-purpose models (e.g., classification of specific types of images or text) into general multipurpose models, such as GPT-3 [35] for text or CLIP/DALL-E for images [36]. These models are mainly based on self-supervised learning with enormous amounts of data; however, in various specific tasks, these general models have outperformed procedures specifically designed for them: although GPT-3 is trained to predict the next word in text, it is also capable of language translation or question answering. As the amount of data generated in biological and, especially, genomic fields grows, the importance of DL methods will continue to increase in the future, as is also the case in other fields.

## Conclusions

**Fig. 2:**
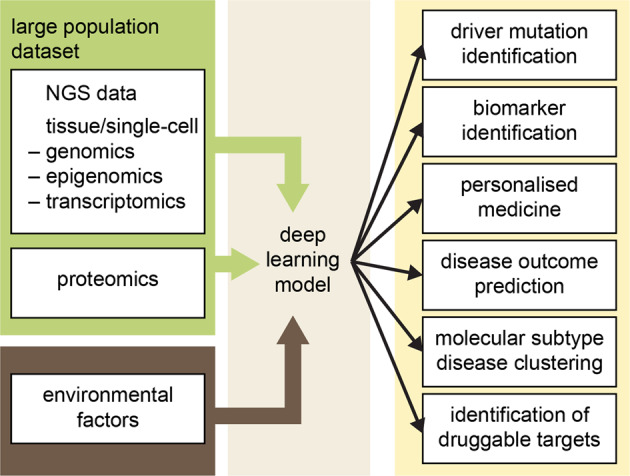
Application of deep learning to the prognosis, diagnosis, and treatment of multifactorial diseases. Multi-omic strategies generating big data accompanied by deep learning may select a meaningful subset of biomarkers and/or genomic alterations to support clinically relevant decisions.

In 2022, we celebrated 200 years since the birth of Johann Gregor Mendel. Although his contributions to science went unrecognized during his lifetime, Mendel pioneered our modern way of doing science based on acquisition of experimental data and their statistical evaluation. The example provided by Mendel shows that what is considered marginal work in one era can become a mainstream research approach in the next era. An integrated strategy that could revolutionize genetic research lies in combining big data with ML or AI, which allows the mining of information hidden in data to identify the genetic models or networks that control various phenotypic traits such as multifactorial diseases. It is also an important tool for developing more effective therapeutic strategies for complex diseases (see Fig. 2).
